# Curcumin-Encapsulated Nanomicelles Improve Cellular Uptake and Cytotoxicity in Cisplatin-Resistant Human Oral Cancer Cells

**DOI:** 10.3390/jfb13040158

**Published:** 2022-09-21

**Authors:** Vijay M. Kumbar, Uday Muddapur, Abdullatif Bin Muhsinah, Saad Ali Alshehri, Mohammed Merae Alshahrani, Ibrahim Abdullah Almazni, Manohar S. Kugaji, Kishore Bhat, Malleswara Rao Peram, Mater H. Mahnashi, Sameer J. Nadaf, Sheetalnath B. Rooge, Aejaz Abdullatif Khan, Ibrahim Ahmed Shaikh

**Affiliations:** 1 Central Research Laboratory, Maratha Mandal’s Nathajirao G. Halgekar Institute of Dental Sciences and Research Centre, Belagavi 590010, India; 2 Dr. Prabhakar Kore Basic Science Research Centre, KLE Academy of Higher Education and Research (KLE University), Belagavi 590010, India; 3 Department of Biotechnology, KLE Technological University, Hubballi 580031, India; 4 Department of Pharmacognosy, College of Pharmacy, King Khalid University, Abha 61441, Saudi Arabia; 5 Department of Clinical Laboratory Sciences, Faculty of Applied Medical Sciences, Najran University, Najran 61441, Saudi Arabia; 6 Chebrolu Hanumaiah Institute of Pharmaceutical Sciences, Guntur 522019, India; 7 Department of Pharmaceutical Chemistry, College of Pharmacy, Najran University, Najran 66462, Saudi Arabia; 8 Sant Gajanan Maharaj College of Pharmacy, Mahagaon 416503, India; 9 Department of Clinical Virology, Institute of Liver and Biliary Sciences, New Delhi 110070, India; 10 Department of General Science, Ibn Sina National College for Medical Studies, Jeddah 21418, Saudi Arabia; 11 Department of Pharmacology, College of Pharmacy, Najran University, Najran 66462, Saudi Arabia

**Keywords:** curcumin nanomicelles, bioavailability, pre-cancer, cisplatin-resistant oral cancer, cytotoxicity

## Abstract

Oral cancer has a high mortality rate, which is mostly determined by the stage of the disease at the time of admission. Around half of all patients with oral cancer report with advanced illness. Hitherto, chemotherapy is preferred to treat oral cancer, but the emergence of resistance to anti-cancer drugs is likely to occur after a sequence of treatments. Curcumin is renowned for its anticancer potential but its marred water solubility and poor bioavailability limit its use in treating multidrug-resistant cancers. As part of this investigation, we prepared and characterized Curcumin nanomicelles (CUR-NMs) using DSPE-PEG-2000 and evaluated the anticancer properties of cisplatin-resistant cancer cell lines. The prepared CUR-NMs were sphere-shaped and unilamellar in structure, with a size of 32.60 ± 4.2 nm. CUR-NMs exhibited high entrapment efficiency (82.2%), entrapment content (147.96 µg/mL), and a mean zeta potential of −17.5ζ which is considered moderately stable. The cellular uptake and cytotoxicity studies revealed that CUR-NMs had significantly higher cytotoxicity and cellular uptake in cisplatin drug-resistant oral cancer cell lines and parental oral cancer cells compared to plain curcumin (CUR). The DAPI and FACS analysis corroborated a high percentage of apoptotic cells with CUR-NMs (31.14%) compared to neat CUR (19.72%) treatment. Conclusively, CUR-NMs can potentially be used as an alternative carrier system to improve the therapeutic effects of curcumin in the treatment of cisplatin-resistant human oral cancer.

## 1. Introduction

Oral cancer is one of the world’s top 18 most common cancers, with 77,713 (2.0) new cases and 177,757 (1.8) cancer deaths worldwide reported annually [1]. Oral cancer accounts for approximately 30% of all cancers in India and ranks among the top three types of cancer in the country, with 119,992 new cases and 72,616 cancer deaths reported annually [2]. It is the leading cause of death in India among men [1]. The common risk factors for oral cancer are betel nut chewing, smoking, and tobacco exposure [1,3].

Conventional cancer treatment involves surgery, radiation, and chemotherapeutic drugs or often multifaceted interventions [4]. Despite promising improvements in the standard therapeutic interventions currently available for patients with oral cancer, there are still a number of issues that need to be resolved. For example, surgical removal can cause permanent disfigurement, altered self-perception, and debilitating physiological consequences. while chemo- and radio-therapies result in significant toxicities, all affecting patient wellbeing and quality of life [5]. Since natural derived compounds are generally less harmful than chemotherapeutics, the scientific community encourages the use of alternative therapies [6]. The secondary metabolism of plants creates a plethora of chemical compounds and serves as a natural laboratory for the drug discovery. In fact, 25% of drugs in common use have plant origins. Their therapeutic potential against different cancers is proved and photochemicals are simple, inexpensive, and less toxic and selectively kill cancer cells without damaging normal cells [7,8].

Curcumin is a phenolic compound and a principal ingredient of the Indian spice, turmeric. It exhibits ample biological activities, such as anti-carcinogenic, anti-inflammatory, antiviral, antimicrobial activities, and pharmacological properties [9]. Pharmacological profiling of curcumin has shown potential anticancer properties, as it exhibits pleiotropic action mechanisms simultaneously influencing multiple pathways [10]. Curcumin targets the transcription factors of cell survival, proliferation, metastasis, angiogenesis, cell cycle regulation-related factors, signaling pathway molecules, kinases, and cytokines, thereby inhibiting the proliferation of cancer cells [11]. It selectively causes tumor cells to induce apoptosis through mitochondria-related intrinsic and death receptor-associated extrinsic pathways [12]. 

In the literature, the role of curcumin against different cancers is well-established using both in vitro and in vivo models. However, still it has failed to be clinically useful as chemotherapy in the treatment of multidrug-resistant cancers owing to its low bioavailability, low solubility, poor pharmacokinetics, high rate of metabolism, and rapid systemic elimination [13]. To overcome these drawbacks, novel nano-drug delivery systems have been developed to enhance the bioavailability of lipophilic drugs. Various novel nanomedicine-based therapeutic drug deliveries, such as liposomes, polymeric nanoparticles (NPs), dendrimers, inorganic nanoparticles, nanoemulsions (NEs), and nanosuspensions, have been designed to potentiate the bioavailability of CUR for anticancer therapy [14,15]. Lin et al. developed the zwitterionic biodegradable DOX-loaded micelles, showing potential antitumor properties [16]. Goto et al., formulated the Gemcitabine loaded polyion complex vesicle (PICsome) encapsulating mesoporous silica nanoparticle showed good targeted cancer chemotherapy [17]. Similarly, Baumann et al. coated DNA on the liposomes for the targeted delivery approaches for cancer treatment [18].

The limitations of these available novel systems include the complex and costly methods of preparation (Solid lipid nanoparticles, polymeric nanoparticles, and liposome), stability problems (liposomes), aggregation, and opsonization in the bloodstream (polymeric nanoparticles), high cytotoxicity, hemolytic properties, non-biodegradable (dendrimers and inorganic nanoparticles), prone to destabilization, temperature fluctuations and even dilution due to a limited amount of surfactant which cannot resist film formed on the large surface area for longer time (nanoemulsions).

Compared with other drug carriers, micelles have the advantage of a very small size (10–100 nm), which is critical for the passive targeting of solid tumors, particularly poorly vascularized tumors. Micelles are nanosized colloidal dispersions prepared from amphiphilic molecules with a hydrophobic tail and a hydrophilic head [19]. Polymeric micelles are macromolecules assembled from block amphiphilic copolymers in aqueous solutions [19]. MPEG-DSPE, 2000 is a derivative of phosphatidylethanolamine and is a widely used nanocarrier for therapeutic drug delivery. It is useful to nanocarriers for attaining prolonged blood circulation time, enhanced stability, and improved encapsulation efficiency [20].

In the present study, we report the preparation and characterization of a novel nanomicellar from 1,2-Distearoyl-sn-glycero-3-phosphoethanolamine-N-methoxy-poly(ethylene glycol 2000) (DSPE-PEG) to enhance the aqueous solubility, improve stability of curcumin and evaluate the anticancer properties and expression of drug resistance genes using a cisplatin-resistant cancer cell line.

## 2. Materials and Methods

### 2.1. Materials

Firstly, p1,2-Distearoyl-sn-glycero-3-phosphoethanolamine-N-methoxy-[poly(ethylene glycol); PEG MW2,000] (DSPE-PEG) was gifted from LIPOID GmbH (Ludwigshafen, Germany). Curcumin (purity > 95% total curcuminoids) was a gift from Kancor Ingredients Ltd., Kerala, India. HPLC grade acetonitrile (purity 99.9%) and methanol (purity 99.8%) were purchased from Fisher Scientific, Mumbai, India. MTT (3-(4,5-dimethylthiazol-2-yl)-2,5-diphenyltetrazolium bromide) (purity 97.5%) and DAPI (4R0,6-diamidino-2-phenylindole) (98%) were purchased from Sigma Aldrich, St. Louis, MO, USA. Dimethyl sulfoxide (99.9%) and paraformaldehyde (purity 95%) were purchased from Qualigens, Mumbai, India. Acridine orange and ethidium bromide were purchased from Himedia Laboratories, Mumbai, India.

### 2.2. Drug-Resistant Cell Lines Development

Drug-resistant cell lines were developed using the previously reported method of Govindan et al. [18]. Briefly, cisplatin-resistant oral cancer sublines (Cis-KB) were developed by repeated exposure of parental cells (KB) with a stepwise dose incremental strategy. The cisplatin’s half-maximal inhibitory concentration (IC_50_) value of cisplatin against the oral cancer cell line was calculated using MTT assay. The KB cell lines were incubated for 24 h with increasing concentrations of cisplatin drugs ranging from IC_6.25_ to IC_50_. After each treatment, surviving cells were cultured in a drug-free medium for 3–5 days and subsequently subjected to the 3rd cycle of the next higher concentration drug treatment. The MTT assay protocol was followed to calculate the IC_50_ values of parental cells and developed resistant cell lines. The resistance index (RI) is the ratio of the IC_50_ value of resistant cell lines to the IC_50_ value of parental cell lines [21].

### 2.3. Preparation of Curcumin Nanomicelles

The Preparation of curcumin nanomicelles (CUR-NMs) was carried out by the film rehydration/reconstitution method with minor modifications [22]. Briefly, curcumin and DSPE-PEG-2000 were solubilized in organic solvent methanol at 180 µg/mL and 1 mM, respectively, in volumetric flasks and vortexed properly to obtain the complete solubilized clear solution. Curcumin and DSPE-PEG solutions were added at suitable ratios (1:1) to round bottom flasks. The solvent was eventually drained under a vacuum at 55 °C and 150 rpm for 30 min using a vacuum rotary evaporator (Rotavapor R210, Buchi, Switzerland). It was purged with argon and kept in a vacuum in dark for overnight incubation. The dried film was rehydrated with 10 mM PBS (pH 7.4), and dissolution of the film was performed by repeated vortexing, accompanied by bath sonication for 5 min.

### 2.4. Characterization of Curcumin Nanomicelles

#### 2.4.1. Curcumin Nanomicelle Size, Size Distribution, and Zeta Potential

A Zetasizer Nano ZS (Malvern Instruments Ltd., Malvern, UK) was used to assess the size and size distribution of CUR-NM formulations using the dynamic light scattering technique (DLS). The zeta potential (ZP) of the CUR-NMs was also quantified by Zetasizer Nano ZS using the M3-PALS method. Using Milli-Q water, a suitable dilution was made, and quantification was carried out at 25 °C by determining the electrophoretic mobility [23].

#### 2.4.2. Morphology and Structure—Transmission Electron Microscopy (TEM)

The morphological features, such as nanomicelle shape and lamellarity, of CUR-NM formulations were characterized by TEM (H-7500, Hitachi, Tokyo, Japan). Appropriate dilution was performed using [milli-Q water. On the surface of the carbon-coated copper grid, one drop of the diluted CUR-NMs sample was coated, and then aqueous uranyl acetate was added as a negative stain. After the completion of grid drying at room temperature, the samples were viewed by TEM at an increasing voltage of 100 kV with a magnification of 40,000× [24].

#### 2.4.3. Entrapment Efficiency

The ultracentrifugation technique was used to determine the entrapment efficiency of CUR [21]. A nanomicelle aliquot was mounted in ultracentrifuge tubes and centrifuged using a Sorvall TMMTX 150 Micro Ultracentrifuge (Thermo Scientific, Mumbai, India) at 40,000× *g* rpm for 3 h at 4 °C. Following centrifugation, free CUR found in the supernatant was collected in separate tubes. Similarly, the dispersion of micelles was lysed with methanol and subjected to sonification to determine the total CUR present in the prepared CUR-NMs. A suitable dilution was conducted, and filtration was also performed with 0.45 nm syringe filters. The quantification of CUR content was performed by using a reported high-performance liquid chromatography (HPLC) method [24]. The percentage entrapment efficiency (% *EE*) was determined as follows:

$$EE\left(\%\right)=\frac{\left(T-S\right)}{T}\times 100$$

where, *T* is the total quantity of CUR in the CUR-NMs solution;

*S* is the quantity of CUR existing in the supernatant,
*T − S* is the quantity of CUR existing within the micelles.

### 2.5. In Vitro Release Study

The dialysis technique was followed to study the in vitro release of CUR previously described by Peram et al., with minor adjustments [24,25]. In short, 1 mL of CUR-NMs was applied to the dialysis bag, and then the dialysis bag was submerged in a flask containing 200 mL of release medium (PBS pH 6.8, 37 °C, and 0.5% (*w*/*v*) SDS). To solubilize permeated curcumin, SDS was added, while SDS addition to the receiving media imitated the in vivo conditions to ensure sink conditions. At a fixed time, the same quantity of fresh medium was substituted whenever an aliquot of 500 μL release medium was extracted and filtered through 0.45 μm membrane filters. A total of A10 μL of the filtrate was injected into the HPLC, and the quantity of curcumin was then obtained and analyzed. Finally, the percentage of curcumin released from micelles over time was plotted.

### 2.6. Cell Uptake Study

The qualitative evaluation of the cellular uptake of CUR micelles was performed by fluorescence microscopy. The cells were seeded in flat-bottomed polystyrene, noncytotoxic, nonpyrogenic, sterilized 24-well culture plates (Nunclon Delta Surface) containing coverslips and kept in a Galaxy 170 R CO_2_ incubator (37 °C, 95% humidity, and 5% CO_2_) overnight. The cells were then treated with CUR and CUR-NMs and incubated for 6 h, followed by washing the wells twice with DPBS. Cell fixation was carried out using paraformaldehyde for 15 min. Cells were then viewed at 40× magnification under a fluorescence microscope (Olympus BX41, New York, USA). Excitation was detected and acquired with the aid of ProRes^®^ Capture Pro software (Jena, Germany) with the 488 nm line of an argon laser and Cur-derived emission fluorescence between 515 nm and 545 nm. Furthermore, we measured the green fluorescence intensity of CUR- and CUR-NM-treated cells [24].

### 2.7. Cytotoxicity

We performed cytotoxicity by an MTT assay. In each well, approximately 5 × 10^3^ cells were seeded in flat-bottomed polystyrene, noncytotoxic, nonpyrogenic, sterilized 96-well culture plates (Nunclon Delta Surface) and kept in a CO_2_ incubator (37 °C temp, 95% humidity, and 5% CO_2_) overnight. The cells were then treated with various concentrations of CUR and CUR-NMs and incubated for 48 h. After completion of incubation, washing wells twice with DPBS was carried out, and 60 µL of the MTT reagent (1 mg/mL in HBSS) was added to all wells, including untreated cells. Furthermore, the plate was incubated for 4 h in a CO_2_ incubator. Then, 100 µL of DMSO was added to all wells, including untreated wells, to dissolve the insoluble dark purple-colored formazan crystals. Using a microplate reader (Bio-Rad, Hercules, CA, USA), the absorbance was recorded at 570 nm, and the following formula was applied to calculate the surviving cells [26],

$$Surviving\;cells=\frac{Mean\;OD\;of\;Test\;Sample\;}{Mean\;OD\;of\;Negative\;Control}\times 100$$

### 2.8. Detection of Mitochondrial Transmembrane Potential

The parent and drug-resistant cells were added at a concentration of 1 × 10^5^ cells per well in flat-bottomed polystyrene, non-cytotoxic, non-pyrogenic, sterilized 12-well culture plate (Nunclon Delta Surface) has cover glass (Cat No-TCP17) and kept in a CO_2_ incubator (37 °C temp, 95% humidity and 5% CO_2_) for overnight. Then, 15 µL of CUR and CUR-NMs were added to the wells and incubated for 12 h. Following incubation, the washing of cells was carried out with DPBS, and a 4% paraformaldehyde solution was used for fixation. After cell fixation, rhodamine 123 dye (5 mg/mL) was added, incubated for 30 min, and observed under a fluorescence microscope at 10× magnification [27].

### 2.9. Dual Staining Assay (Acridine Orange/Ethidium Bromide)

The parent and drug-resistant cells were added at a concentration of 5 × 10^4^ cells per well in flat-bottom polystyrene, noncytotoxic, nonpyrogenic, sterilized 24-well culture plates with coverslips and incubated in a CO_2_ incubator overnight. Then, 15 µL of CUR and CUR-NMs were added to the wells and incubated for 48 h. Following incubation, the cells were rinsed with DPBS, and a paraformaldehyde (4%) solution was used for fixation. Then, a half-hour incubation of 20 μL of the dye mixture ethidium bromide (10 mg/mL) and acridine orange (10 mg/mL) in PBS was performed and examined under a fluorescence microscope at 10× magnification [24].

### 2.10. 4′,6-diamidino-2-phenylindole (DAPI) Staining

The parent and drug-resistant cells were added at a concentration of 5 × 10^4^ cells per well in flat-bottom polystyrene, noncytotoxic, nonpyrogenic, sterilized 24-well culture plates with coverslips and incubated in a CO_2_ incubator overnight. Then, 15 µL of CUR and CUR-NMs were added to the wells and incubated for 48 h. Following the incubation time, cells were rinsed with DPBS, and a paraformaldehyde (4%) solution was used for fixation. After fixation, the cells were covered with DAPI solution at 10 µg per ml for 15 min. Again, the sections were rinsed with DPBS and examined under a fluorescence microscope at 40× magnification [24].

### 2.11. Apoptosis by Flow Cytometer

The parent and drug-resistant cells were added at a concentration of 2 × 10^5^ cells per well in flat-bottom polystyrene, noncytotoxic, nonpyrogenic, sterilized 12-well culture plates (Nunclon Delta Surface) and incubated in a CO_2_ incubator overnight. The treatment IC_50_ value of each sample was 12 h. Following incubation, the cells were rinsed with DPBS. The centrifuge was carried out for 6 min at 450× *g* at 4 °C. After removing the supernatant, an ice-cold 1X binding buffer was added to the cell pellets to a cell concentration of 1 × 10^5^ per mL. Tubes were kept on ice, and 1 μL and μL of annexin V-FITC solution and PI solution were added, respectively. The tubes were gently mixed, held in ice, and further incubated in the dark for 15 min. Add an ice-cold 1X binding buffer of 400 μL. Then, the data were collected from a FACSCalibur (Becton Dickison Biosciences) and evaluated with Flow Jo software (Flow X 10.0.7) [28].

### 2.12. Biocompatibility Assay

The cytotoxicity of CUR, CUR-NMs, and blank micelles were performed against human periodontal fibroblasts (PDF) cells by using MTT assay. An approximately 5 × 10^3^ cells were seeded in a 96-well microtiter plate and maintained at 37 °C in 95% humidity and 5% CO_2_ overnight. The cells were then treated with various concentrations of CUR, CUR-NMs, and blank micelles and incubated for 48 h. The wells were washed twice with PBS. 20 µL of the MTT reagent solution (5 mg mL^−1^ in PBS) was added to each well and plate was incubated for 4 h in the dark. The formazan crystals formed were then dissolved by adding 100 µL dimethyl sulfoxide (DMSO), and absorbance was recorded at 570 nm using microplate reader (Bio-Rad, Hercules, CA, USA) [26].

$$Surviving\;cells=\frac{Mean\;OD\;of\;Test\;Sample\;}{Mean\;OD\;of\;Negative\;Control}\times 100$$

### 2.13. Statistical Analysis

Statistical analysis was carried out using GraphPad PRISM software version 5.1 (GraphPad Software Inc., San Diego, CA, USA). The cell viability assays, gene expression studies, relative fluorescence intensity of cell uptake study and mitochondrial membrane potential study, and apoptosis assays were analyzed by comparison of parental oral cancer and respective cisplatin drug-resistant cancer lines using one-way ANOVA followed by Bonferroni’s multiple comparison procedures. Similarly, we calculated the IC_50_ of all cell viability assays by taking the log of inhibitor vs. response. The difference was regarded as significant when *p* < 0.05, 

## 3. Results

### 3.1. Drug-Resistant Cell Lines Development

**Figure 1:**
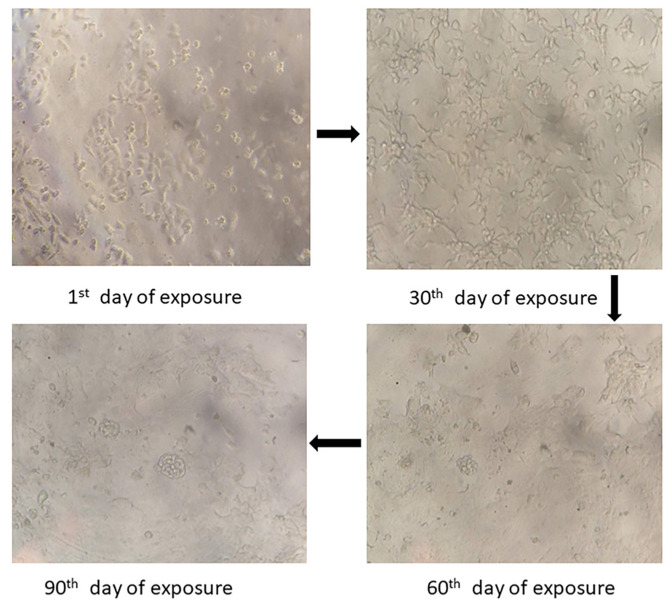
The changes in morphology upon increased concentration treatment with cisplatin were visualized using inverted microscopy at 20× magnification.

A resistance index (RI) greater than or equal to 2 was considered chemoresistance. The IC_50_ values of parental and cisplatin drug-resistant cell lines were 4.52 µg/mL and 14.43 µg/mL, respectively. The developed cisplatin drug-resistant cell line RI was 3.19, confirming that the lines were chemo-resistant. Under an inverted light microscope, both parental and cisplatin drug-resistant cells grew adhering to the bottom. The parental cells (KB) were medium in volume, even in size, with little cytoplasm and a clear nucleolus. The cisplatin drug-resistant cells were larger than the parental cells (KB) cells in volume and triangular or irregular in shape with a markedly enlarged nucleus and cytoplasm, more multiple nucleoli, and slow-growing cells, as shown in Figure 1.

### 3.2. Determination of the Size, Size Distribution, and Zeta Potential of CUR Nanomicelles (CUR-NMs)

**Figure 2:**
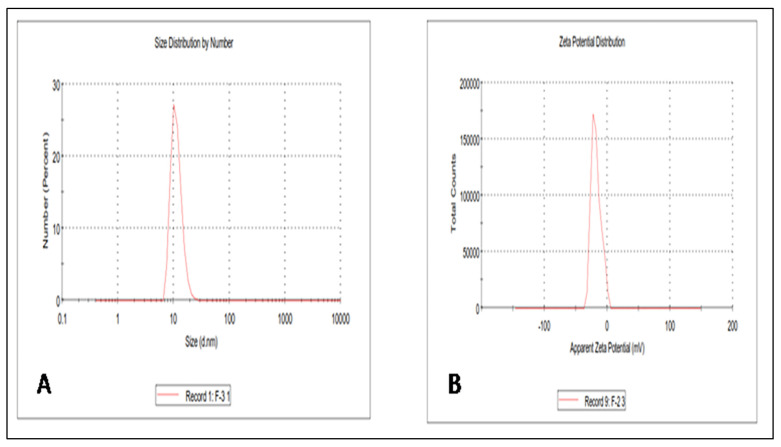
The vesicle size and zeta potential of Curcumin nanomicelles (Cur-NMs). (**a**) size distribution; (**b**) zeta potential distribution.

The size of the nanomicelles is important for taking advantage of the enhanced permeability and retention effect (EPR) for passive targeting ability in the tumor. By using the dynamic light scattering (DLS) method, the CUR-NM size and size distribution were measured. The mean particle size of CUR-NMs was 12.60 ± 4.2 nm, and the PDI was 0.208, as shown in Figure 2. This corroborates that CUR-NMs were nanoscale and homogenous in the population due to their small PDI value. The mean zeta potential of CUR-NMs was −17.5ζ which is considered moderately stable.

### 3.3. Structure and Morphology of CUR-NMs by Transmission Electron Microscopy (TEM)

**Figure 3:**
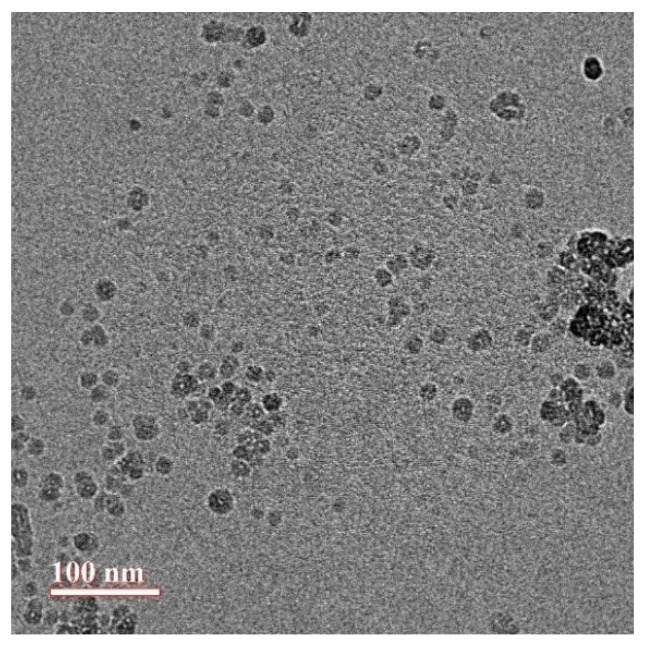
TEM photomicrographs of curcumin nanomicelles (Cur-NMs).

The structure and morphology of CUR-NMs were studied using TEM. The results showed the spherical and homogeneous structure of CUR-NMs, as shown in Figure 3. The TEM photomicrographs of CUR-NMs showed a smaller comparison DLS technique since the dehydration step in TEM analysis can cause the hydrophilic surface to shrink, whereas higher hydrodynamic volume due to solvent influence in the hydrated state is calculated in the DLS method.

### 3.4. Entrapment Efficiency

The entrapment efficiency and content of curcumin within the CUR-NMs was found to be 82.2% and 147.96 µg/mL, respectively, based on calculations described in the methods. 

### 3.5. In Vitro Release Study

**Figure 4:**
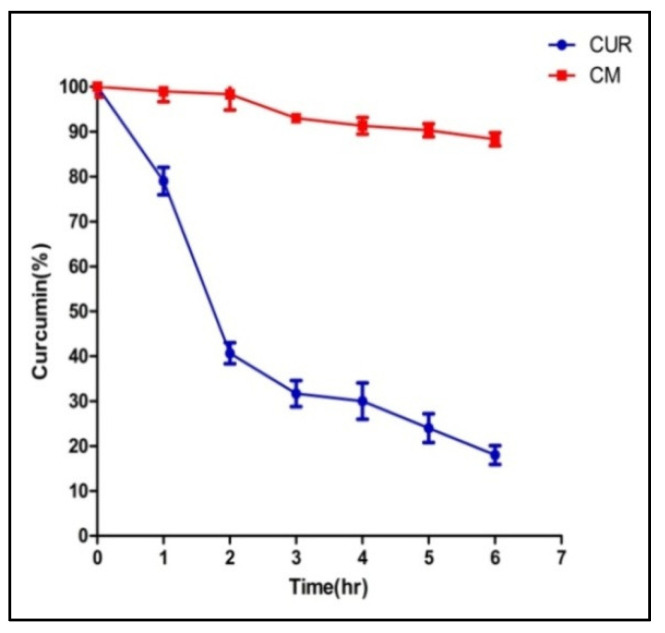
In vitro release profile of curcumin and curcumin nanomicelles (Cur-NMs).

The study of in vitro release supported the controlled release of CUR from the physiological pH of CUR-NMs, as shown in Figure 4. In comparison with native CUR, CUR-NMs released from only 38 ± 2.1% of CUR at 24 h, whereas native CUR released reached 41.5 ± 3.6% at the initial 10 min. The cumulative native CUR release was 85.68 ± 3.21% at 48 h, but in the case of CUR-NMs, it reached 65.43 ± 2.16%. This confirmed that the curcumin in vitro release profile of nanomicelles showed an improved controlled-release property.

### 3.6. Cell Uptake Study

**Figure 5:**
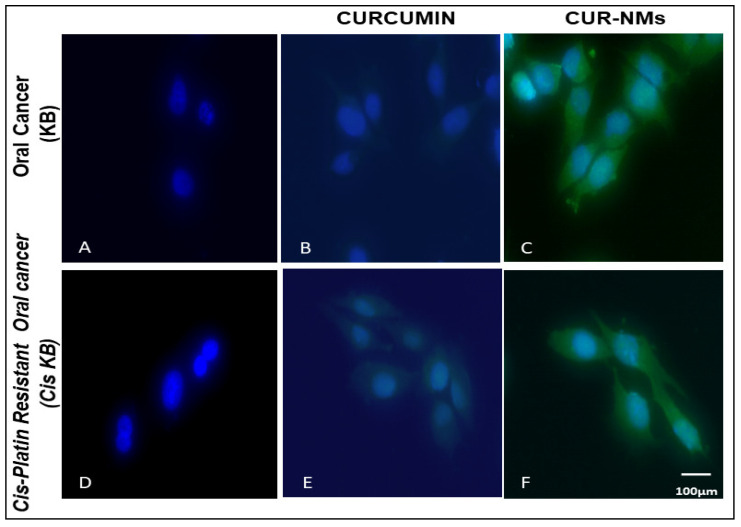
(**A**–**F**) Fluorescence microscopic images showing the cellular uptake of curcumin and curcumin-nanomicelles (Cur-NMs) in an oral cancer cell line (KB) and a cisplatin-drug resistant oral cancer cell line (Cis-KB) after 6 h of incubation. Blue indicates the nucleus of the cells stained with DAPI, and green indicates the presence of CUR.

**Figure 6:**
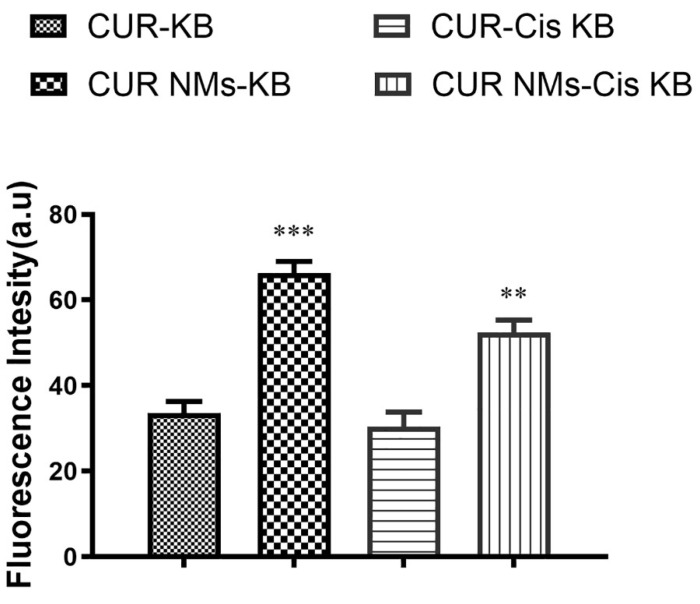
Bar charts showing the green fluorescence intensity (%) of curcumin and curcumin nanomicelles (Cur-NMs) in an oral cancer cell line (KB) and a cisplatin-drug resistant oral cancer cell line (Cis-KB). The differences in significance were specified as *p* < 0.05 (significant), between curcumin and curcumin nanomicelles (CUR-NMs). The difference in significance was specified as ** *p* < 0.01 (moderately sig-nificant), and *** *p* < 0.001 (highly significant).

Cellular uptake experiments were performed on cell lines using fluorescence microscopy to check the mechanism for the improved cytotoxicity of the formulation of CUR-NMs. As shown in Figure 5, the significantly increased green fluorescence intensity of CUR-NMs in parental (KB) and cisplatin drug-resistant (Cis-KB) cell lines compare to native CUR confirmed the intracellular uptake of CUR-NMs. There were significant increases in the intensity of green fluorescence of CUR-NMs in comparison to CUR, as shown in Figure 6.

### 3.7. Cytotoxicity

**Figure 7:**
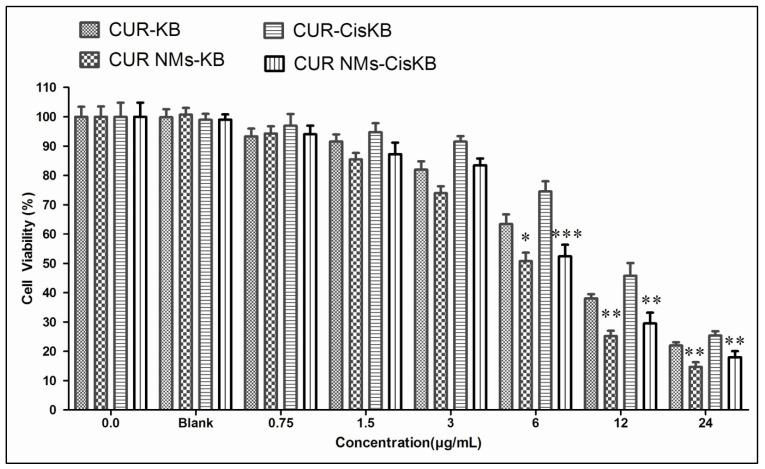
In vitro cytotoxic effect of curcumin and curcumin nanomicelles (CUR-NMs) on an oral cancer cell line (KB) and a cisplatin-drug resistant oral cancer cell line (Cis-KB) for 48 h. Significant differences were specified as *p* < 0.05 (significant), between curcumin and curcumin nanomicelles in both cell lines. The difference in significance was specified as * *p* < 0.05 (significant), ** *p* < 0.01 (moderately sig-nificant), and *** *p* < 0.001 (highly significant).

The MTT assay was performed to test the cytotoxic effects of CUR and CUR-NMs. The IC_50_ values for CUR treatment were 8.84 µg/mL and 11.48 µg/mL in the oral cancer cell line (KB) and cisplatin-drug resistant oral cancer cell line (Cis-KB), respectively. Similarly, the IC_50_ values for CUR-NMs treated at 48 h were 6.01 µg/mL and 11.48 µg/mL, respectively. Only at lower concentrations, i.e., treatment with 1.56 µg/mL and 3 µg/mL, CUR-NMs caused cytotoxicity but did not show significant cell death compared to native CUR. Other concentrations of CUR-NMs exhibited a significantly (*p* < 0.01) increased cytotoxic effect than CUR (Figure 7). This may be attributed to the controlled release of CUR from NMs contributing to the continuous exposure of the drug to tumor cells, which has positive effects on firm anticancer potential.

### 3.8. Effects of Curcumin on Mitochondrial Membrane Potential (ΔΨ) 

**Figure 8:**
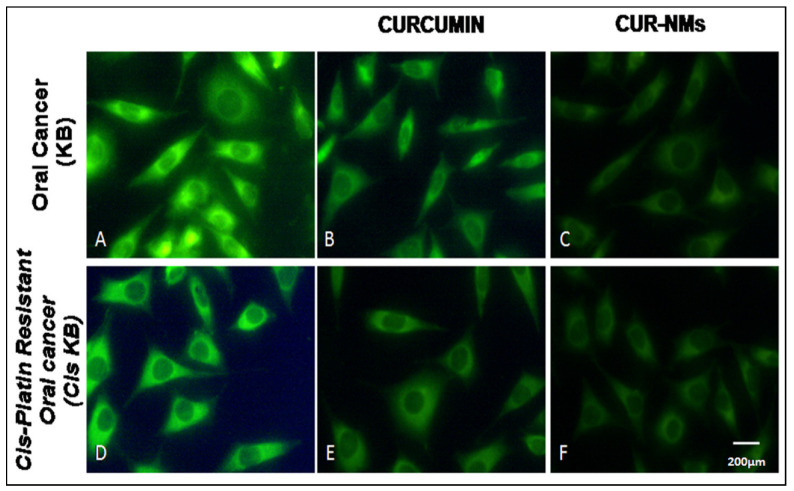
(**A**–**F**) Fluorescence microscopic images showing the mitochondrial transmembrane potential of curcumin and curcumin nanomicelles (Cur-NMs) in an oral cancer cell line (KB) and cisplatin-drug resistant oral cancer cell line (Cis-KB) after 8 h of incubation. Cells treated with CUR and CUR NMs decrease green staining due to changes in MMP(Magnification-40×).

**Figure 9:**
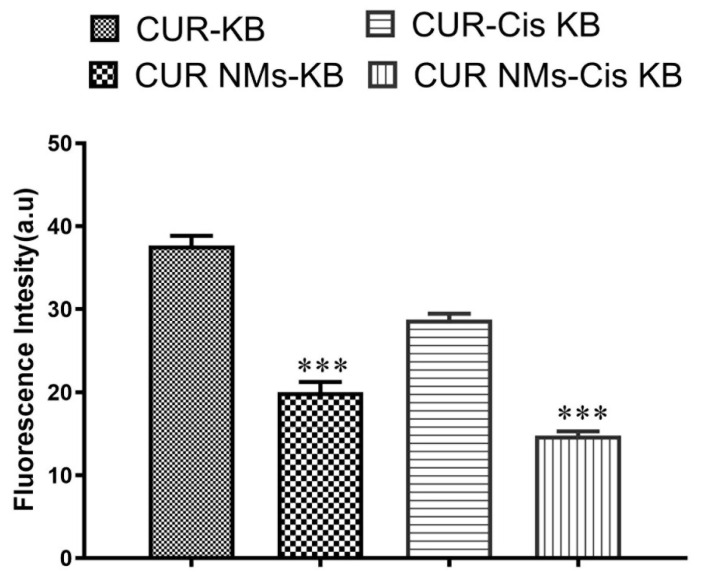
Bar charts showing the green fluorescence intensity (%) of curcumin and curcumin nanomicelles (Cur-NMs) in an oral cancer cell line (KB) and a cisplatin-drug resistant oral cancer cell line (Cis-KB). The difference in significance was specified as *** *p* < 0.001 (highly significant) between curcumin and curcumin nanomicelles (CUR-NMs).

The loss of mitochondrial membrane potential (ΔΨ) of cells signifies the early stage of apoptosis. We evaluated the impact of CUR and CUR-NMs on the loss of mitochondrial membrane potential (ΔΨ) in cells, which was assessed by a rhodamine-123 dye. After overnight incubation, CUR-NM-treated cells showed a significant change in mitochondrial membrane potential. There is often diminished green fluorescence in comparison with CUR-treated cells that emit high-intensity green fluorescence, indicating the collapse of mitochondrial membrane potential both in an oral cancer cell line (KB) and cisplatin-drug resistant oral cancer cell line (Cis-KB), as shown in Figure 8. The significant decrease in green fluorescent intensity (%) of CUR-NMs treated cells compared to native curcumin is shown in Figure 9.

### 3.9. Double Staining (Acridine Orange/Ethidium Bromide)

**Figure 10:**
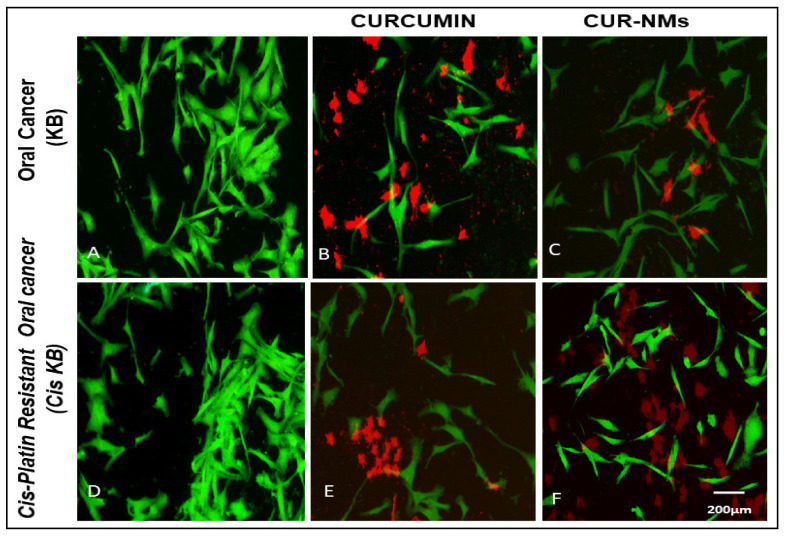
(**A**–**F**) Fluorescence microscopic images showing the live and dead cell assay of curcumin and curcumin nanomicelles (Cur-NMs) in an oral cancer cell line (KB) and cisplatin-drug resistant oral cancer cell line (Cis-KB) after 24 h of incubation. Cells treated with CUR and CUR NMs showed a dark orange-red color. The live cells demonstrated green fluorescence and dead cells exhibited dark-orange red fluorescence (Magnification-10×).

It is evident that in the control, live cells were stained with acridine orange and fluoresced green in color with spindle-shaped, adherent, and higher monolayer cell confluence. CUR-NMs and CUR treatment at a concentration of 15 µg/mL for a time of 48 h induced cell death. CUR-treated cells showed red fluorescence due to ethidium bromide staining, with some cells becoming round in morphology. CUR-NM-treated cells emitted a deep red color, indicating remarkable cell death due to the presence of ethidium bromide, and almost all cells became circular with fewer cells, as shown in Figure 10.

### 3.10. DAPI

**Figure 11:**
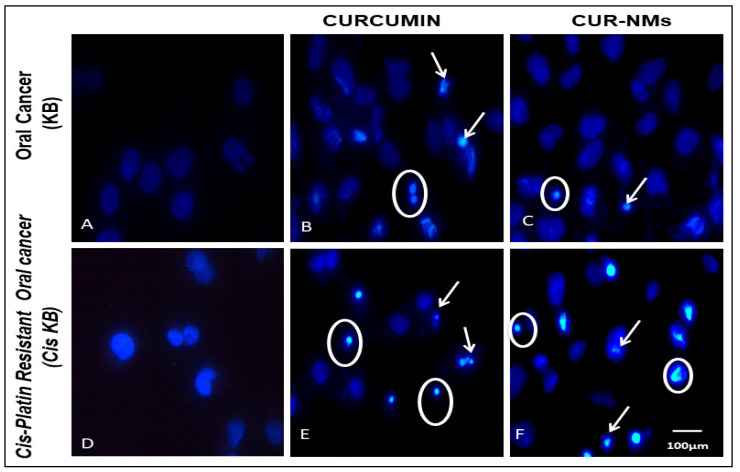
(**A**–**F**) Fluorescence microscopic images showing the Apoptotic nuclear changes observed in an oral cancer cell line (KB) and cisplatin-drug resistant oral cancer cell line (Cis-KB) after treatment with curcumin and curcumin nanomicelles (Cur-NMs) for 24 h of incubation. Cells treated with CUR and CUR NMs showed small nuclei with bright chromatin condensation, blebbing, nuclear fragmentation, and apoptotic body formation. Arrow represents chromatin condensation and nuclear shrinkage, nuclear fragmentation. Circles represent nuclear blebbing and apoptotic bodies (Magnification-40×).

The apoptosis-inducing potential of CUR was determined by DAPI staining. Untreated cells were without any cellular or morphological changes. However, when treated with CUR, few cells exhibited chromatin condensation with highly significant cell apoptosis. Similarly, CUR-NM-treated cells showed nuclear fragmentation; chromatin condensation and blebbing of the nucleus were observed with cell apoptosis, which was highly significant (*p* < 0.001) when compared to control cells, as shown in Figure 11.

### 3.11. Apoptosis

**Figure 12:**
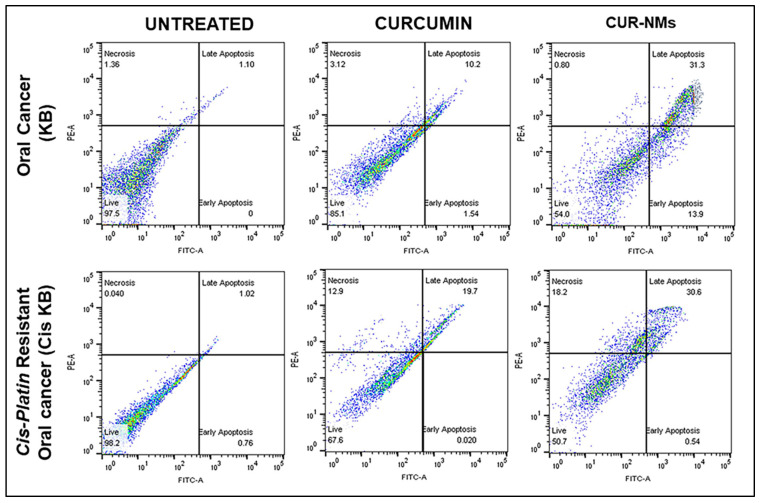
Histograms of flow cytometric analysis displaying the effect of curcumin and curcumin nanomicelles (Cur-NMs) on apoptosis in an oral cancer cell line (KB) and cisplatin-drug resistant oral cancer cell line (Cis-KB) after treatment for 48 h using Annexin V-FITC and PI staining. The illustrative figures are separated into four quadrants showing the population of live (annexin V−/PI−), early apoptotic (annexin V+/PI−), late apoptotic (annexin V+/PI+), and necrotic (annexin V+/PI+) cells.

**Figure 13:**
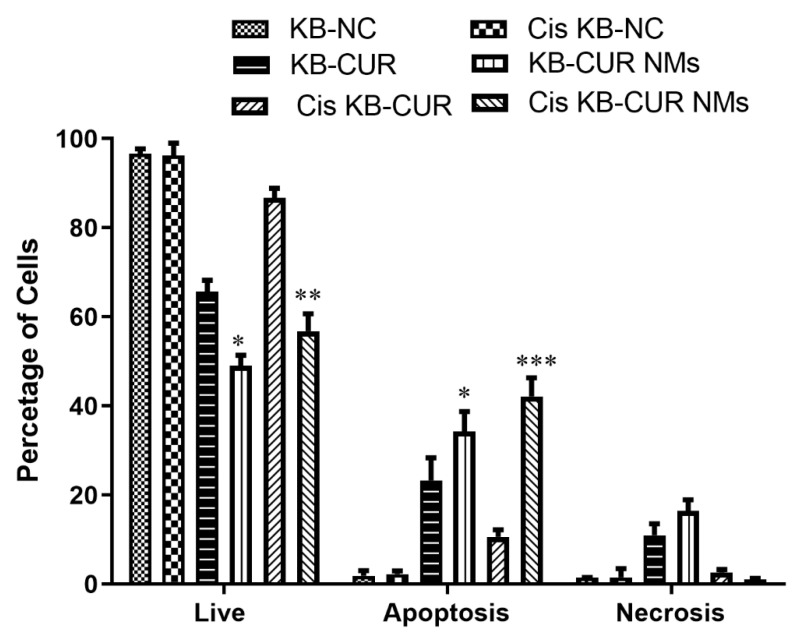
Bar charts showing the percentage of live cells, early apoptosis, late apoptosis, and necrotic cells in an oral cancer cell line (KB) and cisplatin-drug resistant oral cancer cell line (Cis-KB) after treatment with curcumin and curcumin nanomicelles (Cur-NMs) for 48 h. The difference in significance was specified as * *p* < 0.05 (significant), ** *p* < 0.01 (moderately significant), and *** *p* < 0.001 (highly significant) between curcumin and curcumin nanomicelles (CUR-NMs).

Quantitative apoptosis analysis was carried out using FACS the following staining with Annexin V and PI staining kits. The cells were then exposed to the corresponding formulations and incubated for 24 h. The cells were then stained with Annexin V and PI, as shown in Figure 12. The untreated control oral cancer (KB) cells showed 97.5%, 1.10%, and 1.36% viability, apoptosis (early and late), and necrosis of cells, respectively. The live-cell percentage significantly decreased to 67.6% in CUR-treated cells (oral cancer) and 50.7% in CUR NM-treated cells (oral cancer) in comparison to untreated oral cancer cells. Similarly, the percentage of apoptosis (early and late) significantly increased to 19.72% and 31.14% in CUR- and CUR NM-treated oral cancer cells (KB), respectively, in comparison to untreated oral cancer cells, as shown in Figure 13.

The untreated control cells of cis-platin-resistant oral cancer (Cis-KB) had 98.2%, 1.78%, and 0.40% viability, apoptosis (early and late), and necrosis of cells, respectively. The live-cell percentage significantly decreased to 85.1% in CUR-treated cells (cisplatin-resistant oral cancer) and 54.0% in CUR NM-treated cells (cisplatin-resistant oral cancer) in comparison to untreated cisplatin-resistant oral cancer cells (Cis-KB). Similarly, the percentage of apoptosis (early and late) significantly increased to 11.74% and 35.10% in CUR and CUR NM-treated cis-platin-resistant oral cancer (Cis-KB) cells, respectively, in comparison to untreated cells. The remarkable apoptosis potential of CUR NMs was attributed to its increased intracellular uptake and controlled release of drug from the nanoparticles both in oral cancer (KB) and cisplatin-resistant oral cancer (Cis-KB), as shown in Figure 13.

### 3.12. Biocompatibility Assay

**Figure 14:**
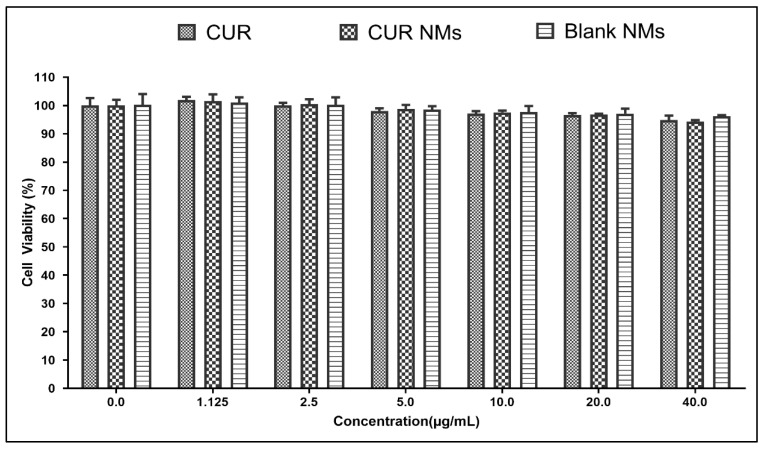
Biocompatibility assay of CUR, CUR-NMs, and blank micelles on PDL cells.

The MTT assay results concluded that at the CUR, CUR-NMs, and blank micelles the cell viability were ≥90% as shown in the Figure 14.

## 4. Discussion

Oral cancer is the world’s 9th most widespread cancer [3]. Oral cancer accounts for approximately 30–40% of all cancer sites in India because of smoking and chewing tobacco habits. The potential therapeutic approaches are surgery, radiation therapy, chemotherapy, and amalgamation, which are used for the treatment of diseases. Stasio et al. case control study showed that there is no significance observed in the psychiatric symptoms [29]. Lucchese et al. suggest that vulval, vaginal and gingival (VVG)-LP lesions is rare condition reported in the case reports might be precise diagnostic criteria oral lichen planus [30]. The treatment of OL and OSF with oral curcumin and lycopene seems to be both efficient and safe [31]. Health professionals and scientists are working on advancing the early diagnosis and prevention of oral cancer [32]. In the latest research, it has been revealed that the presence of CSCs is responsible for the failure of currently available therapies and appears to be one of the primary causes of treatment failure. CSCs are responsible for increased tumorigenicity; enhanced resistance to chemotherapy and radiation therapy; aggressive migration, invasion, and metastasis; and local recurrence, distant metastasis, and therapeutic resistance [33].

### 4.1. Drug-Resistant Cell Lines Development

A resistant index (RI) greater than or equal to 2 was considered as chemoresistance. In the present study, the development of cisplatin drug resistance in the cell line RI was 3.19, confirming that the lines were chemo-resistant. Drug-resistant cell lines have become necessary in vitro modeling systems, which may promote the perception of the significance of the fundamental tools of clinical anti-cancer drug resistance. Cancer cells with a developed resistance to a broad diversity of anticancer drugs have been developed and tested for different cancer types [34]. Additionally, natural herbal products have been screened as anticancer drugs, as they have shown multiple targets of carcinogenesis to overcome drug resistance.

### 4.2. Characterization of Curcumin Nanomicelles (Curcumin Nanomicelle Size, Size Distribution, and Zeta Potential)

A nanotechnology approach has been followed to develop CUR micelles to enhance bioavailability, pharmacokinetics, stability, and sustained drug release. The nanomicelles were prepared by a lipid-film method. Zetasizer and TEM analysis showed that the mean particle size of CUR-NMs was less than 100 nm. In 2017, Huang et al. prepared gefitinib-loaded nanomicelles with CD133 aptamers that were approximately ~20 nm in size with a low PDI of less than 0.2, suggesting narrow and homogenous populations of nanomicelles. The higher negative zeta potential (approximately −20 mV) of nanomicelles has high circulation stability [35]. Otilia et al. revealed that camptothecin- micelles had a mean diameter of 13.8 ± 0.4 nm with a small size distribution [36]. Sok Bee Lim et al. developed a sterically stabilized phospholipid micelle formulation of human glucagon-like peptide-1 as a unique anti-inflammatory drug delivery system. The resulting micelles had a mean size of 13.6 ± 3.1 nm with a single size peak [37]. The mean particle size of CUR-NMs is less than 100 nm for oral absorption, which is helpful to the distribution of proleptic tissue and the enhanced permeability and retention effect (EPR) for the passive targeting ability of micelles in the tumor [38]. ZP is the charge on the surface of the CUR-NMs and is an important parameter used to evaluate the physical stability of the formulation. Irrespective of charge, the higher the ZP value is, the greater the long-term stability of CUR-NMs, thus preventing aggregation between the same charges of CUR-NMs due to electrostatic repulsion. Overall negative surface charge on nanomicelles could be attributed to the typical conjugation of PEG to distearoylphosphatidylethanolamine (DSPE) by a carbamate linkage which gives a net negative charge on the phosphate moiety at physiological pH [39]. Previously reported DSPE-PEG2000-based nanoemulsions also showed negative surface charges [40].

### 4.3. Entrapment Efficiency

Entrapment efficiency corresponds to the percentage of drugs combined within the nanomiceller formulation. It is useful to predict the drug-holding capacity of the drug delivery system. Based on calculations, we found that the entrapment efficiency of curcumin within the CUR-NMs was >85%. Huang et al. in 2017 prepared gefitinib-loaded nanomicelles with a drug-loading efficiency >85%; thus, the lipid film rehydration method was successful in the entrapment of curcumin in nanomicelles [35].

#### 4.3.1. In Vitro Release Study

The controlled release of CUR from the CUR-NMs at physiological pH was verified by an in vitro release analysis. Huang et al. in 2017 prepared gefitinib-loaded nanomicelles and gefitinib-loaded nanomicelles with CD133 aptamers, which showed faster drug release in PBS with plasma (10%) than in PBS. Approximately 50% of gefitinib release was observed in the initial 12 h of incubation, and in the next 36 h of incubation, more than 70% of gefitinib was released, suggesting sustained drug release for 48 h. This rapid degradation of curcumin was mainly induced within a small time by hydrolysis and biotransformation of curcumin into glucuronide and sulfate conjugates [35]. Such elevated degradation and instability characteristics were also similarly demonstrated by Mohanttly et al., who claimed that almost 90% of curcumin undergoes degradation after 6 h of incubation in PBS solution. Therefore, we could conclude that our curcumin micelles with great improvement in the stability of curcumin might have potential applications in cancer therapy. Although curcumin was embedded in a hydrophobic center, the rate of degradation was slow, leading to increased oral absorption and therefore oral bioavailability [41]. This regulated release of curcumin from CUR NMs indicated that its usefulness as a passive targeted drug delivery may reduce healthy tissue exposure and increase the retention of anticancer drugs in tumor sites.

#### 4.3.2. In Vitro Cytotoxicity Assay

MTT is a colorimetric test used for the evaluation of the cytotoxic activity of nanoformulations against cancer cell lines. Mitochondrial succinate dehydrogenase enzyme in viable cells was used to convert MTT reagent into formazan sugar crystals dissolved in DMSO, and dark purple coloration was measured using a microplate reader. Although MTT reduction can occur mostly in metabolically active cells, the intensity level is a measure of cell viability. In the current research, the antiproliferative activity of CUR and CUR-NMs against oral and cisplatin drug-resistant cancer cell lines was experimentally proven. The present research has shown experimental proof of antiproliferative CUR and CUR-NM activity against oral cell lines and cisplatin-resistant cancer cells. CUR showed good cancer cell inhibition, as indicated by the IC_50_ value, which ranged from 8.84 to 11.48 µg/mL. Chang et al. stated that CUR-encapsulated filled PLGA nanoparticles (Cur-NPs) at 10 µM did not decrease cell viability, whereas, at 20, 40, and 80 µM, they significantly decreased cell viability in cisplatin-resistant oral cancer cell lines [42]. Chen et al. revealed that cetuximab and CUR showed dose-dependent cytotoxicity, but CUR showed more cytotoxicity than cetuximab. Even the synergistic effects of cetuximab and curcumin were a significant increase in cytotoxic effects in cisplatin-resistant oral cancer cell lines [43].

### 4.4. Cell Uptake Study

The cellular uptake of CUR and CUR-NMs were carried out using fluorescence microscopy. As CUR has autofluorescence properties with excitation and emission ranges between 425 nm and 470 nm, respectively, it emits green fluorescence that can help to observe the uptake of CUR inside the cell using a fluorescence microscope. In our study, CUR-NM-treated cells showed strong and consistently distributed fluorescence observed both inside and outside of the nucleus after 4 h of incubation compared to native CUR. There was a significant increase in the intensity of green fluorescence of CUR-NMs in comparison to CUR, confirming the intracellular uptake of curcumin. The fluorescence dye-binding gefitinib-loaded nanomicelles with CD133 aptamer-treated cells showed significantly increased fluorescence intensity related to fluorescence dye-binding gefitinib-loaded nanomicelles and blank micelles [35]. Wang et al., in 2012 prepared curcumin-filled stearic acid-chitosan polymeric micelles, which showed evidence of significantly higher green fluorescence intensity compared to free curcumin [44]. This may be due to the absorption of CUR-NMs via endocytosis, providing an effective route for the drug to be transported across the cell membrane, especially to drug-resistant tumor cells. Numerous reports have indicated that CUR nanoparticles display improved cell uptake and deposition in many cancer cell lines. The improved intracellular uptake of CUR-NMs in our study might be due to their nanosized micelles. Nanoparticles with a small size of less than 100 nm have the potential to readily reach the cell and are even capable of moving through the nuclear membrane and accumulating in the nucleus of the cell. Thus, small nanoparticles are useful for the passive targeting of tumor tissue by improved permeation and retention effects [38].

Effects of curcumin on the loss of mitochondrial membrane potential (ΔΨ) of the oral cancer cell line (KB) and cisplatin-drug resistant oral cancer cell line (Cis-KB).

The mitochondrial-mediated pathway is one of the major apoptosis signaling pathways induced by CUR in many cancer cells. Mitochondrial membrane potential collapse is an early step in the apoptotic cascade, as confirmed by numerous studies [45]. The collapse in the mitochondrial membrane potential was observed after the increase in CUR concentration treatment in the current research. Similar results were shown by Jin-bo Wang et al., who showed that CUR induces mitochondria-mediated apoptosis in the human colon cancer cell line HT-29 [46]. A decrease in MMP damage was associated with a decline in P-gp expression and the repression of cell resistance when we were treated with curcumin. The quick loss of ΔΨm, organellar enlargement and discharge of cytochrome C are known to be the key features of mitochondrial dysfunction that trigger caspase pathways and induce apoptotic cell death [45].

### 4.5. Double Staining (Acridine Orange/Ethidium Bromide)

A dual (AO/EB) fluorescent staining test was used to determine the cell viability depending on the integrity of the cell membrane. Viable cells have an undamaged cell membrane, while dead cells have a damaged cell membrane [24,47]. In live cells, acridine orange diffuses through the cytoplasmic membrane and intercalates with the cytoplasmic and nuclear RNA of living cells, generating strongly homogenous green fluorescence. In the case of dead cells, both the cell membrane and nuclear membrane are damaged, so ethidium bromide enters the cells and intercalates into the base pairs of the double helix, generating bright orange/red fluorescence [24,48]. In this assay, we used a combination of two fluorescent dyes (AO and EB) that variably labeled live and dead cells. Our study clearly showed live cells with green fluorescence and dead cells with yellowish to red fluorescence. The treatment of cells with CUR caused cell death, resulting in dark orange-red fluorescence with more round cells. Similarly, the CUR-NM treatment of cells showed a dark orange-red color, indicating substantial cell death, and almost all cells became circular with fewer cells. AO/EB staining showed that curcumin at concentrations of 5 µmol/L, 10 µmol/L, and 20 µmol/L for 24 induces apoptosis in a multidrug-resistant human gastric carcinoma cell line [49]. Peram et al. 2018 reported that CUR- and optimized curcumin ethosome-treated A375 cells emitted a dark orange-red color under fluorescence microscopy, indicating substantial cell death. Untreated cells were green in color, with consistent intensity indicating live A375 cells [24].

### 4.6. DAPI

Changes in nuclear morphology are a hallmark of apoptosis. In the early apoptotic process of chromatin condensation, nuclear shrinkage occurs. First, fragmentation of the nucleus into larger molecular weight fragments (50–300 kb) and then into low molecular weight fragments (180 bp) occurs. DAPI is a blue fluorescent, minor groove-binding probe for DNA, and it is simple to visualize nuclear DNA in both living and fixed cells. It diffuses through the cell membrane and attaches with the double-stranded DNA minor groove. DAPI staining has been used to assess the changes in nuclear morphology and gross cell morphology [24,50]. Untreated cells did not show any nuclear morphology, whereas, in CUR-treated cells, nuclear fragmentation and chromatin condensation were observed. Similarly, CUR-NM-treated cells showed nuclear fragmentation, chromatin condensation and blebbing of the nucleus, indicating that the cells underwent apoptosis. Chang et al. showed that curcumin-loaded PLGA nanoparticles (Cur-NPs) induce apoptotic cell death in cisplatin-resistant CAR human oral cancer cells [42]. In 2018, Peram et al., reported that CUR- and OPT-CUR-ETH-treated A375 cells showed nuclear fragmentation; chromatin condensation and blebbing of the nucleus indicated that the cells underwent apoptosis. When untreated, cells showed green color with uniform intensity, live A375 cells were observed [24].

### 4.7. Apoptosis by Flow Cytometry

Apoptosis is a dynamic physiological mechanism triggered by unique morphological and biochemical changes in the nucleus and cytoplasm during cell self-destruction [51]. Under normal physiological conditions, choline phospholipids such as phosphatidylcholine and sphingomyelins are seen on the external leaflet, whereas amino phospholipids such as phosphatidylserine and phosphatidylethanolamine are found on the inner side of the cytoplasmic surface of the lipid bilayer. Phosphatidylserine (PS) is exposed to the outer leaflet of the membrane during apoptosis and is detected by fluorochrome-tagged 36 kDa anti-coagulant protein annexin-V in the presence of calcium ions. Propidium iodide is a red fluorescent nucleic acid binding dye that easily enters cells with cell membrane damage, whereas it is impermeable to live and early apoptotic cells. Annexin V-FITC/PI staining techniques were utilized to detect the survival, early apoptosis, late apoptosis, and necrosis of the cells using FACS. Therefore, annexin V-FITC/PI staining differentiated cells into 4 quartiles: quartile-1 annexin V−/PI− was live, quartile-2 annexin V+/PI− was early apoptotic, quartile-3 annexin V+/PI+ was late apoptotic, and quartile-4 annexin V−/PI+ was necrotic. CUR is a natural herbal product that shows good anticancer properties by targeting multiple pathways. CUR is a well-recognized natural molecule that induces apoptotic cell death in different types of tumor tissues, including breast cancer, lung cancer, colorectal cancer, pancreatic cancer, prostate cancer, gallbladder cancer, and hepatocellular carcinoma [24]. We used the annexin V-FITC/PI staining technique to investigate the cell death mechanism of CUR and CUR-NMs in a drug-resistant cancer cell line. The apoptotic cell percentages were 18.69% and 33.6% in CUR and CUR NMs, respectively. Enhanced apoptosis was observed approximately 1.5 times more for CUR NMs in comparison to curcumin. The remarkable apoptosis potential of CUR NMs was attributed to its elevated intracellular uptake and controlled release of curcumin from the nanoparticles.

### 4.8. Biocompatibility Assay

The biocompatibility is crucial in determining if it is safe for human usage. Blank micelles, CUR, and CUR-NMs did not exhibit any cytotoxicity effects on the viability of healthy cells in the current research effort. This demonstrates its biocompatibility for use in treating oral cancer [26].

## 5. Conclusions

In summary, CUR-NMs enhanced cytotoxic potential with improved cellular uptake in cisplatin-resistant human oral cancer. CUR-NMs trigger apoptotic cell death through changing the mitochondrial membrane potential. CUR-NMs are promising for development as a novel medicine against cisplatin-resistant human oral cancer. In the future, we are planning to develop the targeted nanoparticle drug delivery system as Curcumin nanomicelles with CD133 or CD44 aptamers for enhanced delivery of Curcumin.

## Data Availability

All the data has been included in the results section of this article.

## Abbreviations

Polymeric nanoparticles	NPs
Nanoemulsions	NEs
Polyion complex vesicle	PICsome
1,2-Distearoyl-sn-glycero-3-phosphoethanolamine-N-methoxy-[poly(ethylene glycol); PEG MW2,000	DSPE-PEG-2000
3-(4,5-dimethylthiazol-2-yl)-2,5-diphenyltetrazolium bromide	MTT
6-diamidino-2-phenylindole	DAPI
Dimethyl sulfoxide	DMSO
Acridine orange and Ethidium bromide	AO and ETBR
Cisplatin-resistant oral cancer sublines	Cis-KB
Curcumin Nano micelles	CUR-NMs
Half-maximal inhibitory concentration	IC_50_
Resistance Index	RI
Zeta Potential	ZP
Transmission Electron Microscopy	TEM
High-Performance Liquid Chromatography	HPLC
Entrapment Efficiency	EE
Dulbecco’s Phosphate Buffered Saline	DPBS
Human periodontal fibroblasts	PDF
enhanced permeability and retention effect	EPR
dynamic light scattering	DLS
mitochondrial membrane potential	MMP
vulval, vaginal and gingival (VVG)-	VVG
Cancer Stem Cells	CSCs
